# Our Daily Experience

**Published:** 1890-12

**Authors:** Wm. Steinrauf

**Affiliations:** St. Charles, Mo.


### OUR DAILY EXPERIENCE.

Wm. Steinrauf, M. D., St. Charles, Mo.

I think the physicians of our school should pay more attention to the committing to paper their daily experience, and after sifting the same, occassionally send short articles of cures made to the journals. I know some of our best prescribers often hesitate about writing down their experience. They see the journals are filled up anyhow from month to month and so they let matters go. Still others shun the notoriety and have a false delicacy in regard to contributing to the journals, thinking their productions of no value.

But, as in the case of a fire, even the most timid give the alarm, even the weakest feel called upon to help, so it should be in our case. False Homœopathy is openly taught and practiced in our ranks, professed homœopathists are openly declaring that there is but very little, if any, difference between our school and the old system. So it behooves us, it behooves all the true and faithful followers of the master to sound the trumpet of alarm, to be outspoken, to openly declare their convictions and show by their work the true and only method of cure. Do like the children of Israel, "with one hand do the work and with the other hand hold the shield." Practice pure Homœopathy and guard the threshold to prevent false prophets from entering.

Clinical Case.—J. K., aged seventy-seven years, was severely attacked with erysipelas of the face and scalp during the epidemic of la grippe last March, and as he is of a scrofulous constitution, and has had chronic eczema of the nose for over twenty years, the outlook was rather discouraging. The swelling had begun on the nose and having a homœopathic family case, he had doctored himself. But when the whole face was attacked and the scalp sore, puffy, and highly inflamed he consented to have a physician. The pulse was one hundred and twenty and temperature one hundred and four. But as he never had been much dosed in his life, always using our remedies, besides being of a determined will, we hoped for the best.

Ferr-phos.$^{200x}$, followed by Natr-sulph.$^{200x}$ cured the patient within six days. Dilute alcohol was applied every hour. What was peculiar in the case is that the eczema of the nose of twenty years' standing was also cured. The eczema disappeared shortly afterward. This cure was two years ago. No erysipelas and no eczema to this date.

